# On the Rupture of the Eyeball

**Published:** 1876-02

**Authors:** F. C. Hotz

**Affiliations:** Chicago


### EXAMINER.

Vol. XXXIII. — FEBRUARY, 1876. — No. 2.

### Original Communications.

### ON THE RUPTURE OF THE EYEBALL.

By F. C. HOTZ, M.D., Chicago.

The force required to destroy with a blunt instrument the continuity of the sclerotic, the firmest and strongest tissue of the eyeball, is so great that usually some or all of the other structures are more or less involved. But for these complications, a rupture of the sclerotic would not be a very serious accident; for it tolerates lesions very well, as we can daily observe in the kind healing of the incisions made intentionally into the sclero-corneal junction for the removal of cataracts, and of wounds inflicted accidentally upon the sclerotic proper. I once saw a young surgeon in an European eye-infirmary, operate for an inveterate convergent strabisumus; and while severing the tendon of the internal rectus, he was unfortunate or unskillful enough to cut into the sclerotic, also wounding the choroid and retina, making a small button-hole through which the vitreous humor could be seen. The eye was bandaged at once and the patient was kept in bed for a few days, after which time the wound was healed without any serious damage resulting from the injury. These lesions were incised wounds, it is true; but there are good reasons to suppose a lacerated wound of the sclerotic, unattended by any serious complications, will heal kindly too, although its edges be not quite so smooth. As the rupture occurs always at a place far remote from where the blunt instrument strikes and bruises the eyeball, the borders of the split do not suffer any contusions, and the disturbance of their nutrition is not great enough to prevent a healing by first union if the wound is properly closed.

It makes very little difference whether the conjunctiva over the rupture is torn or not, nor does this give any measure of the degree of the force which caused the accident.

It is not meant that the blow must have been less forcible and injurious to the interior structures of the globe, when the conjunctiva is not torn, than when it is.

I have seen a subconjunctival rupture of a globe whose inner structure was thoroughly mashed. The end of a billiard cue had been thrust in the eye with a terrible result. The sclerotic was burst over the upper half of the eyeball, a profuse bleeding occurred into the eye, forcing out through the wound all the vitreous humor and the greater part of the retina, and crowding the iris and lens to the posterior surface of the cornea. The conjunctiva was not torn, but it was raised from the sclerotic by the accumulation of blood and vitreous humor. The patient did not consent to the extirpation of his demolished eye; and to alleviate his suffering the conjunctiva was cut into and a large quantity of blood evacuated. Here the blow was thoroughly destructive to the eye, and yet the conjunctiva was not involved. And in regard to the healing, the subconjunctival rupture has no advantage over the open wound; nay, I might say, the latter offers a better chance, because it can be cleansed nicely, and closed accurately by a suture. And a good healing depends, in the first place, on a good apposition of the borders of the wound.

The location of the rupture is of some consequence only when it passes through the ciliary ring, because then it is, even without any complications, a very serious lesion on account of the well known dangerous character of inflammation of the ciliary body. But when the rent is in front of, or behind, the ciliary region, the location itself has no decisive weight in determining the question whether the lesion will be fatal to the eye. This depends solely and entirely on the complications usually attending the rupture. And these complications are as different in seriousness as the physiological characters of the various structures composing the contents of the eyeball. We observe an effusion of blood into the anterior chamber, a detachment of the iris from its ciliary border, or a prolapse of the iris; the lens may be dislocated or forced out of the eyeball through the wound, a portion of the vitreous humor may have escaped or blood may fill the whole posterior chamber in place of the vitreous humor; the retina may be detached from the choroid, either in consequence of concussion or by an effusion of blood between the two membranes; and a portion of the choroid and retina may protrude through the rent in the sclerotic. A number of these complications involve the most vital parts of the eye, which then must be regarded as irretrievably lost.

It is unfortunate that these cases constitute the majority, among the lesions in question. No wonder, therefore, if many consider a rupture of the globe as equivalent to the loss of this organ, and believe nothing short of the extirpation of the eye is to be done, although this operation is not always indicated. If we can positively ascertain that the vital parts of the eye have seriously suffered; if we are certain the eye will never be of any good use, but become a source of continual suffering to the patient, we are right in advising its immediate removal. But in many cases we are unable, on the first examination, to estimate the full magnitude of the injury; the globe is burst, and the anterior chamber filled with blood, so much so that we are utterly ignorant about the condition of the iris, lens and other parts; they may be seriously damaged, or they may possibly have escaped any great injury; we cannot tell. To sacrifice the eye under such conditions I would call a very unjustifiable proceeding, and I mention this because I am well aware of the fact that under just such circumstances the excision of the eye has been advised and performed by ophthalmic surgeons. There is no reason for being in such haste. We can not run any risk by waiting until the obscuration of the anterior chamber is removed, so that we can realize the actual extent of the injury and shape our treatment accordingly, or until such symptoms arise as positively prove the hopeless disorganization of the inner structure of the eye and demand its extirpation. And although many eyes have then to be removed, and one might say the immediate excision would have saved the patient a good deal of pain and time, as it has to be done after all, I would rather bear this blame than to be charged with having sacrificed an eye which might have been saved by a less hasty and more judicious treatment. I like, in doubtful cases, always to give the patient the benefit of the doubt. And how well this expectant treatment is sometimes rewarded, the following cases may illustrate. They have been under my observation long enough to warrant the permanency of the result.

1. *Rupture of the sclerotic; loss of vitreous humor; suture seven days after the accident; recovery.*

On August 11, 1874, A. N., aged thirty years, while hunting, received so heavy a blow on his head by the explosion of his gun, that he was stunned for several minutes. When recovered, he found the right eyelids swollen to such an extent that he could not open the eye. This swelling of the lids subsided under the use of cold water, and on the third day after the accident, he could open his eye again to find that sight though somewhat impaired, was still retained. As the eye continued to be red and tender, he used, on the advice of a physician, a lotion of acetate of lead.* From this time the inflammation steadily increased in intensity, and the visual power steadily decreased at the same rate.

On August 18, when I examined the eye, it could scarcely distinguish light from darkness. The ocular conjunctiva was intensely reddened, and studded with ecchymoses; over the inner half of the globe it was torn, the rent beginning at six millimetres from the inner margin of the cornea, and extending horizontally backwards for seven millimetres. In the same direction, though less extensively, the sclerotic, choroid and retina were ruptured, and the gaping wound was filled with a gray pluck of protruding vitreous humor. The cornea showed a faint parenchymatous haziness, the aqueous humor was somewhat turbid and colored by effused blood, the pupil of a medium width, immovable and not quite circular, because a small portion of the inner margin was less retracted than the remainder of the iris, and so formed a little tongue protruding into the pupillary space. This irregularity was not caused by posterior synechiæ or irido-dialysis, and it disappeared in a few days. The lens was transparent, but the vitreous humor was densely clouded and did not permit of any illumination of the interior of the eye. The tension of the eyeball was markedly diminished and the upper ciliary region was very sensitive to the touch.

This case resembled, in several points, the two cases of gaping wounds of the sclerotic which George Lawson has recorded in his "Injuries of the Eye, Orbit and Eyelids;" in the one case the wound was closed by a suture on the third day, in the other on the eighth day after th

* It is strange, but nevertheless true, that whenever physicians have to prescribe for "sore eyes," they almost invariably give a lead lotion! If they only could bear in mind that this is, generally speaking, a dangerous eye water and must be used with the greatest precaution! Every text book and every lecturer teaches this, and still they prescribe it with criminal carelessness.

accident, and in both a very gratifying result was obtained. True, Lawson's were *incised* wounds, while ours was a *rupture* of the sclerotic, produced at a somewhat unusual place,* by a sudden, violent blow upon the eye. But as this blunt force struck the eyeball at another point than where its tunics were torn, and as the edges of the wound were not contused but smooth and straight, the chances for its healing when united by a suture, were just as favorable as in the cases quoted above, unless the violent blow had established a deep-seated intra-ocular hæmorrhage, or a detachment of the retina. Such complications, however, could not be thought of, since the patient had affirmed, quite positively, that on the third day after the injury he could still see pretty well. The slight dimness of his sight then was due to the turbidity of the aqueous humor into which a little blood was effused, and the subsequent failure of his vision could well be accounted for by the cloudiness of the vitreous substance which followed the injudicious use of irritating lotions.

The wound was freed from the vitreous protrusion, and its lips were united by a single suture of finest silk. The eye was closed and covered by a moderately firm bandage; the patient was kept in bed in a dark room. On Aug. 21, the suture was removed and the wound remained closed by a fine linear scar; the eye ball appeared fuller, the tenderness of the ciliary region had disappeared entirely, and the patient could already count my fingers at a distance of two feet by candle light. By Aug. 28, the cornea and aqueous humor had become perfectly clear; the pupil, then circular, of normal size, responded readily to the light; the diffuse cloudiness of the vitreous had consolidated itself into a number of dark horizontal streaks; the fundus oculi, though still veiled, could be seen; the tension of the globe was normal, and vision

* "Unusual," because these rents from such a source are known, as a rule, to occur in the anterior part of the sclerotic, and to be near and parallel to the margin of the cornea.

$\frac{20}{200}$. From this time on, I examined the eye repeatedly to confirm a steady decrease of the opacities in the vitreous body; they became fewer in number and shorter, contracting themselves, as it were, toward the place where the sclerotic had been ruptured. On Dec. 30, vision was $\frac{20}{50}$; the exterior of the eye was normal in every respect and so was its interior, excepting a minute conical opacity which marked the seat of the lesion of the ocular tunics. And this improvement was not ephemeral but lasting; at least I found it so about a year ago, when I examined the eye the last time.

2. *Subconjunctival rupture of the sclerotic; prolapse of the iris; recovery.*

On Nov. 7, 1873, J. P. H., 66 years old, presented himself with the following history: Two evenings previously he was in a billiard hall to witness a game, when a billiard ball bounced off the table and struck the old gentleman's left eye. He felt a sharp pain and could see no more with that eye. On the day of his first visit, the pain was very moderate; the upper ciliary region, however, was very sensitive to the touch. There was some tumefaction of the lids and considerable redness and swelling of the conjunctiva; the cornea transparent, but the anterior chamber filled with blood, so much so, that nothing could be ascertained in regard to the pupil and iris. About two millimetres from the upper margin of the transparent cornea, and parallel with it, the conjunctiva was raised up by a dark bluish substance 10 millimetres in length and $1\frac{1}{2}$ millimetres in width. By November 11, the blood effused into the anterior chamber was re-absorbed, and the full extent of the lesion could be realized. The iris exhibited a large defect in its upper half, as if a broad iridectomy had been performed. The dark blue mass lying under the conjunctiva, was the upper portion of the iris protruding through a long subconjunctival rupture of the sclero-corneal border. The refractive media were transparent, and the fundus oculi did not show any anomaly. During strict functional rest, enforced by the use of atropia and a bandage, the eye quickly recovered of the traumatic inflammation. All signs thereof had faded away by Dec. 4, when the sight was $\frac{20}{50}$ (vision of the right eye being $\frac{20}{40}$). The hernia of the iris having shriveled down to a thin atrophic tissue and the scar in the sclero-corneal junction being well consolidated, the above described abnormal elevation of the ocular conjunctiva had made room again for the natural smoothness and normal curvature of the tunics. Only at the inner angle of the wound the scar showed a slight distension and attenuation of the tissue, and fearing that this might lead to a staphylomatous or "cystoid" expansion, I punctured it (December 16) with a broad needle, and excised the little thin flaps. The aqueous humor, of course, was all drawn off; but after a few days the anterior chamber was restored. No further bulging of the cicatrix took place; and up to the present writing, the patient has been using the restored eye with comfort.

